# Marcadores de remodelado óseo, monitorizar el cambiante hueso

**DOI:** 10.1515/almed-2024-0005

**Published:** 2024-02-21

**Authors:** Nerea Varo

**Affiliations:** Bioquímica, Clínica Universidad de Navarra, Pamplona, España

La transición al bipedalismo jugó un papel crucial en la evolución y es una característica distintiva del ser humano que nos diferencia de nuestros ancestros primates. Definitivamente, caminar sobre dos piernas tenía ventajas, pero el esqueleto humano sufrió adaptaciones morfológicas sustanciales, como la curvatura en S de la columna o cambios en la pelvis para soportar la carga.

El esqueleto óseo es un tejido altamente especializado en continuo remodelado durante toda la vida. En un adulto, cerca del 20 % del tejido óseo es remodelado anualmente. Este proceso se logra gracias a la acción coordinada de osteoclastos y osteoblastos. Cuando se rompe el equilibrio entre ambos procesos, los huesos se vuelven más porosos. La primera descripción de la osteoporosis se atribuye al anatomista francés Joseph Guichard Duverney (1648–1730) en su obra póstuma “Tratado sobre enfermedades óseas”. El término “osteoporosis” fue acuñado por el médico francés Lobstein, en 1833, para los huesos porosos que observaba en las autopsias. Actualmente la osteoporosis se define como “una enfermedad esquelética caracterizada por una resistencia ósea disminuida, lo que predispone a un riesgo aumentado de fractura”. Por lo tanto, la osteoporosis viene determinada por la resistencia ósea, que refleja la combinación de densidad y calidad ósea.

La osteoporosis es la enfermedad metabólica ósea más frecuente pero su prevalencia es de difícil estimación por ser asintomática hasta que aparece la fractura, por ello recibe el nombre de epidemia silente. El último informe de la International Osteoporosis Foundation (IOF) describe que, en 2019, 25,5 millones de mujeres y 6,5 millones de hombres tenían osteoporosis en la Unión Europea, Suiza y Reino unido y ocurrieron 4,3 millones de nuevas fracturas por fragilidad [1]. En nuestro país la prevalencia se estima en unos 3 millones de personas de las cuales casi el 80 % son mujeres. La aparición de fracturas por fragilidad es la consecuencia directa de la osteoporosis y resultan en una notable reducción en la calidad de vida, un elevado coste social por la dependencia que provocan y un alto coste económico. De hecho, en España, el coste directo de las fracturas osteoporóticas fue de 1.813 millones de euros y la carga económica de la enfermedad supone un 3,8 % del presupuesto sanitario.

El diagnóstico de la osteoporosis se realiza de acuerdo a los criterios establecidos por la OMS en 1994 basados en la medida de la densidad mineral ósea y expresado como el número de desviaciones estándares que la densidad mineral ósea de un individuo se aleja de los valores medios de la población adulta joven normal del mismo sexo [2]. Sin embargo, el uso de la densitometría ósea como única herramienta diagnóstica y de seguimiento en la osteoporosis, presenta varias limitaciones entre las que destacan que ofrece información cuantitativa del hueso y no cualitativa, que la sensibilidad y valor predictivo de fractura son bajos y que pequeños cambios en la densidad mineral ósea tardan en reflejarse densitométricamente.

Por ello, se han explorado otras alternativas siendo una de ellas los marcadores de remodelado óseo, productos liberados a la circulación sanguínea durante la formación y resorción ósea, pudiendo así evaluarse la tasa de estos dos procesos. Tradicionalmente se han clasificado como marcadores de formación y de resorción ósea. La fosfatasa alcalina fue el primero de estos marcadores que se introdujo en la práctica clínica en 1929. El método para su determinación fue descrito primeramente por King y Armstrong, modificado por Ohmori, Bessey, Lowry y Brock, y finalmente optimizado por Hausamen y cols. Las técnicas actuales se basan en modificaciones de estos métodos.

Al inicio de los años 80, a la par que se produjo un aumento del interés de los clínicos por las enfermedades metabólicas óseas, se empezaron a desarrollar los ensayos más específicos de hueso. La osteocalcina fue uno de los primeros en estudiarse encontrando que reflejaba la formación de osteoide medido en la biopsia ósea. Los años 90 supusieron una gran expansión del desarrollo de nuevos ensayos específicos de hueso. Se introdujeron ensayos con anticuerpos contra la región N-mid de osteocalcina y radioinmunoensayos para la fosfatasa alcalina ósea con muy baja reactividad contra la isoforma hepática, que se utilizaron para identificar respondedores frente al tratamiento con alendronato. Los siguientes fueron los métodos para entrecruzamiento de piridinolina, piridinolina y desoxipiridinolina en orina encontrándose que la excreción aumentada se asociaba a la tasa de pérdida ósea tras la menopausia. La automatización llegó con el desarrollo los métodos para medir el telopéptido C-terminal del colágeno tipo I (CTX) y el propéptido N-terminal del procolágeno tipo I (PINP) en suero por inmunoensayo.

El entusiasmo suscitado con las atractivas características de los marcadores de remodelado óseo, ha estado a menudo contenido debido a la heterogeneidad de ensayos disponibles y a su variabilidad preanalítica, lo que ha hecho que no se hayan implementado de manera amplia en la práctica clínica. El grupo de trabajo conjunto de marcadores óseos de la IOF junto a la International Federation of Clinical Chemistry (IFCC) recomendaron utilizar PINP y CTX en sangre como marcadores óseos de referencia en estudios observacionales y de intervención, y en la monitorización del tratamiento antiosteoporótico [3]. También se reconocía la necesidad de estandarización o armonización de los ensayos comerciales para generar datos comparables y poder utilizarse en clínica y en investigación. El comité del metabolismo óseo de la IFCC (IFCC C-BM) está trabajando para preparar materiales internacionales de referencia. Y a nivel, clínico se está colaborando con la IOF para establecer intervalos de referencia y niveles de decisión universalmente aceptables.

En cuanto a su uso clínico, las guías más importantes, la RACGP (Royal Australian College of General Practitioners), la ESCEO-IOF (European Society for Clinical and Economic Aspects of Osteoporosis-International Osteoporosis Foundation), la NOGG (National Osteoporosis Guideline Group), (North American Menopause Society), la ES (Endocrine Society), y el ACOG (American College of Obstetricians and Gynecologists) coinciden en un uso relativamente restringido del uso de marcadores óseos en centros especializados únicamente con el propósito de monitorizar el tratamiento. En nuestro país, la SEIOMM (2022), en línea con los anteriores, indica que los marcadores de remodelado óseo no son necesarios de manera sistemática en el diagnóstico de la osteoporosis, pero son útiles para evaluar la respuesta terapéutica si se miden en condiciones estandarizadas.

La National Bone Health Alliance (NBHA) ha publicado recomendaciones dirigidas a laboratorios, fabricantes de reactivos y clínicos sobre preparación del paciente y manejo de muestras para reducir la variabilidad preanalítica [4]. Se revisaron los factores controlables e incontrolables relacionados con el paciente para facilitar la interpretación y la recogida de muestras. Las muestras para CTX deben recogerse sistemáticamente en ayunas por la mañana por su ritmo circadiano. Se prefiere el plasma EDTA por la mayor estabilidad de la muestra. Las condiciones de recogida de muestras para el PINP son menos críticas, ya que tiene una variabilidad circadiana mínima y no se ve afectado por la ingesta. Sin duda, la adopción de procedimientos estandarizados de manipulación de muestras y preparación de pacientes reduce significativamente la variabilidad preanalítica controlable. Las variables no controlables (edad, sexo, embarazo, inmovilidad, fractura reciente, comorbilidades, fármacos antiosteoporóticos u otros medicamentos, etc.) también deben tenerse en cuenta en la interpretación de los marcadores de remodelado óseo. La adopción con éxito de estas recomendaciones requiere la estrecha colaboración de diversas partes involucradas desde los laboratorios, a la comunidad médica, incluyendo también a los fabricantes de reactivos.

El presente número monográfico pretende abordar estos retos que han ido surgiendo para los especialistas de laboratorio. Se revisarán los factores que influyen en la variabilidad de los marcadores de remodelado óseo y también, el hueso como órgano endocrino productor de moléculas reguladoras en otros tejidos. La fractura osteoporótica es una complicación no clásica de la diabetes y ha sido generalmente ignorada quizá por su complejo abordaje diagnóstico y terapéutico. Por ello, se revisará el efecto de los fármacos antidiabéticos sobre el metabolismo óseo, así como la asociación de los niveles de osteocalcina con la densidad mineral ósea y polimorfismos del gen del receptor de la vitamina D en la diabetes tipo 1 y 2. También se presenta un estudio sobre el metabolismo óseo en niños con sobrepeso/obesidad.

Dado que la osteoporosis y las fracturas por fragilidad son frecuentes y hay tratamientos efectivos, la mayoría de los pacientes son preferentemente atendidos en Atención primaria, derivando al especialista los casos más complejos. No existe especialidad para el manejo de patología ósea y los pacientes son atendidos según la Comunidad Autónoma a través de especialidades que incluyen medicina interna, traumatología, ginecología, endocrinología y reumatología. El abordaje de la patología ósea requiere formación específica y esta amplia variación en la atención especializada dificulta la consistencia en el cuidado del paciente. El Laboratorio Clínico por su carácter trasversal tiene una posición privilegiada para promover la atención sistemática, protocolos coordinados, estandarización de la fase preanalítica, solicitud de pruebas, ayuda en la interpretación de los resultados de marcadores, etc. Los especialistas de laboratorio debemos atender estas responsabilidades con rigor, estudio y enfoque científico. Además, se generan grandes volúmenes de resultados de manera rutinaria en todos los laboratorios, que pueden de la manera adecuada utilizarse para generar evidencia científica, hipótesis y planear futuros estudios de intervención como ha sugerido la ESCEO.

Nuestro estilo de vida actual conducirá a adaptaciones en el esqueleto desde una perspectiva evolutiva que ahora desconocemos. Mientras tanto, si sabemos que superar los retos anteriormente planteados sin duda redundará en mayor beneficio del sobrecargado sistema sanitario, los clínicos y de las personas con alto riesgo de fractura o ya fracturadas.
